# An Unexpected Spontaneous Motion-In-Depth Pulfrich Phenomenon in Amblyopia

**DOI:** 10.3390/vision3040054

**Published:** 2019-10-22

**Authors:** Alexandre Reynaud, Robert F. Hess

**Affiliations:** McGill Vision Research, Department of Ophthalmology, McGill University, Montreal, QC H3G 1A4, Canada; robert.hess@mcgill.ca

**Keywords:** Pulfrich phenomenon, interocular delay, stereopsis, motion in depth, amblyopia

## Abstract

The binocular viewing of a fronto-parallel pendulum with a reduced luminance in one eye results in the illusory tridimensional percept of the pendulum following an elliptical orbit in depth, the so-called Pulfrich phenomenon. A small percentage of mild anisometropic amblyopes who have rudimentary stereo are known to experience a spontaneous Pulfrich phenomenon, which posits a delay in the cortical processing of information involving their amblyopic eye. The purpose of this study is to characterize this spontaneous Pulfrich phenomenon in the mild amblyopic population. In order to assess this posited delay, we used a paradigm where a cylinder rotating in depth, defined by moving Gabor patches at different disparities (i.e., at different interocular phases), generates a strong to ambiguous depth percept. This paradigm allows one to accurately measure a spontaneous Pulfrich phenomenon and to determine how it depends on the spatio-temporal properties of stimulus. We observed a spontaneous Pulfrich phenomenon in anisometropic, strabismic, and mixed amblyopia, which is posited to be due to an interocular delay associated with amblyopic processing. Surprisingly, the posited delay was not always observed in the amblyopic eye, was not a consequence of the reduced contrast sensitivity of the amblyopic eye, and displayed a large variability across amblyopic observers. Increasing the density, decreasing the spatial frequency, or increasing the speed of the stimulus tended to reduce the observed delay. The spontaneous Pulfrich phenomenon seen by some amblyopes was variable and depended on the spatio-temporal properties of the stimulus. We suggest it could involve two conflicting components: an amblyopic delay and a blur-based acceleration.

## 1. Introduction

The binocular viewing of a fronto-parallel pendulum with a neutral density (ND) filter placed over one eye results in the illusory tridimensional percept of the pendulum following an elliptical orbit in depth, the so-called Pulfrich phenomenon [1]. This is explained as the consequence of the processing delay between the two eyes introduced by the luminance reduction [2,3,4,5]. The underlying mechanism might involve either changes in the pure disparities over time for disparity sensors [6,7,8,9] or changes to sensors that encode motion/disparity conjointly [10,11,12,13].

Some stereo-deficient persons are able to experience the Pulfrich phenomenon [14]. Furthermore, a small percentage (approximately 4%) of mild anisometropic amblyopes who have rudimentary stereopsis experience a spontaneous Pulfrich phenomenon during normal viewing [15]. This is assumed to be due to a delay in the processing of the information coming from the amblyopic eye because of an increased retinocortical transmission time from their amblyopic eye, or because of prolonged neural integration at retinal or cortical sites [16,17,18,19,20]. This delay is observed in the pupillary response to contrast gratings of the amblyopic eye [21,22]. Therefore, it could also be a consequence of the lower contrast gain in the amblyopic eye [23], because a contrast difference between the two eyes also induces a Pulfrich phenomenon that is assumed to be the result of a contrast-dependent neural delay [24]. The spontaneous Pulfrich phenomenon seen by some amblyopes could be due to either a lowpass temporal deficit or the reduced contrast sensitivity of the amblyopic eye. Indeed, McKee et al. [25] observed a delay correlated with interocular contrast sensitivity difference in amblyopia. The purpose of our study was to assess and characterize this delay. Is it fixed or variable? On what does it depend?

To assess and measure the interocular delay (IOD), we used a paradigm where a cylinder rotating in depth is defined by moving Gabor patches of different interocular phases, generating strong to ambiguous depth percepts [24,26,27]. This paradigm allows one to manipulate independently the spatio-temporal properties of the patches to determine their influence on perceived motion-in-depth. Firstly, we assessed the spontaneous Pulfrich phenomenon in amblyopia, and particularly its intersubject variability. Secondly, we manipulated the statistical visual properties of the stimulus to determine if parameters such as density, spatial frequency, and temporal frequency of the stimulus influenced the perceived phenomenon.

## 2. Methods

### 2.1. Participants

Eight mild amblyopic subjects (two anisometropes, two strabismic, four mixed amblyopes; four males, mean age 35.6 ± 12.4 years) with residual stereoscopic vision participated in this experiment. Subject details are reported in Table 1. Among the amblyopic subjects we screened, these patients were the only ones able to perform the task. They approximately represent one-third of the amblyopic subjects screened. This research was approved by the Ethics Review Board of the McGill University Health Center (protocol code 1996-1806) and was performed in accordance with the ethical standards laid down in the Code of Ethics of the World Medical Association (Declaration of Helsinki). Written informed consent was obtained from all subjects.

### 2.2. Apparatus

The equipment used was the same as in Reynaud and Hess, 2017 [24]. Stimuli and experimental procedures were programmed with Matlab R2015a (© the MathWorks, Natick, MA, USA) using the Psychophysics toolbox (version: 3.0.12—flavor: beta) [28,29,30] running on a Linux Mint operating system, on an Apple MacPro computer with an Nvidia GeForce 8800 GT graphics card. Dichoptic presentation was achieved using a polarized passive wide 23-inch three-dimensional (3D)-Ready light-emitting diode (LED) monitor ViewSonic V3D231 in interleaved line stereo mode, so that the two images to the two eyes were presented at the same time but on different scanlines at a refresh rate of 60 Hz. The monitor was gamma-corrected with a mean luminance of 100 cd·m^−2^ and a resolution of 1920 × 1080 px, placed at a viewing distance of 90 cm, in a dim-lit room. Subjects wore passive polarized ViewSonic 3D glasses that generated a luminance reduction of approximately 60% and a crosstalk of 1% [31].

### 2.3. Stimuli

**Figure 1:**
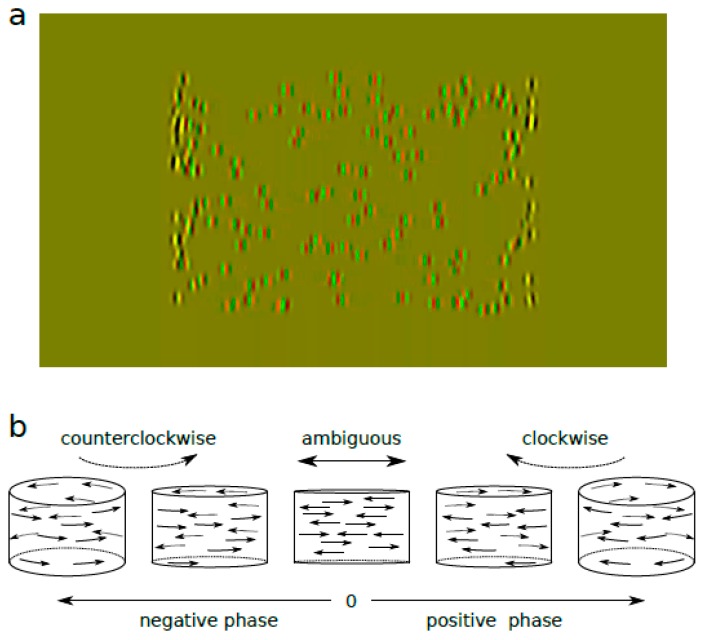
Stimuli. (**a**) Red/green anaglyph representation of the stimulus. It is composed of 200 Gabor patches oscillating horizontally and presented dichoptically. (**b**) The phase difference in the oscillation of the Gabor patches between the two eyes generates a percept of a motion-defined cylinder rotating in depth. If the phase difference is negative, the cylinder is seen rotating anticlockwise. If the phase difference is 0°, the percept is ambiguous with Gabor patches moving to the left and to the right in the same plane. If it is positive, the cylinder is seen rotating clockwise. Figure adapted from Reference [24].

The stimulus was a structure-from-motion defined rotating cylinder of 18° width and 12° height, consisting of Gabor patches oscillating horizontally with a sinusoidal speed (Figure 1a, supplementary movies). The stimulus was presented dichoptically for 800 ms. In order to generate percepts of the cylinder rotating in depth, Gabor patches were displaced between the two eyes as a function of the interocular phase difference in their oscillation. This interocular phase difference was consistent between all Gabor patch trajectories and was varied from trial to trial to generate strong to ambiguous percepts (Figure 1b) [24,26,27]. Gabor patch initial positions were randomized in each trial. In the base condition, we used 200 Gabor patches at 80% contrast, each of size 0.15° sigma, 2.85 c/d spatial frequency, and 1.36 octave bandwidth with random phase, with a sinusoidal angular speed (degrees of phase) of 18°/s.

### 2.4. Procedures

The subjects’ task was to report within a block design paradigm whether they saw the cylinder rotating clockwise or anticlockwise as a function of the interocular phase. Phase was picked with a constant stimuli procedure within [−1.5, −0.75, −0.375, −0.1875, −0.0938, −0.0469, −0.0234, 0 0.0234, 0.0469, 0.0938, 0.1875, 0.375, 0.75, 1.5]° with 10 repetitions in each block. One condition was tested per block. Each block was repeated three times in a counterbalanced way.

### 2.5. Data Analysis

**Figure 2:**
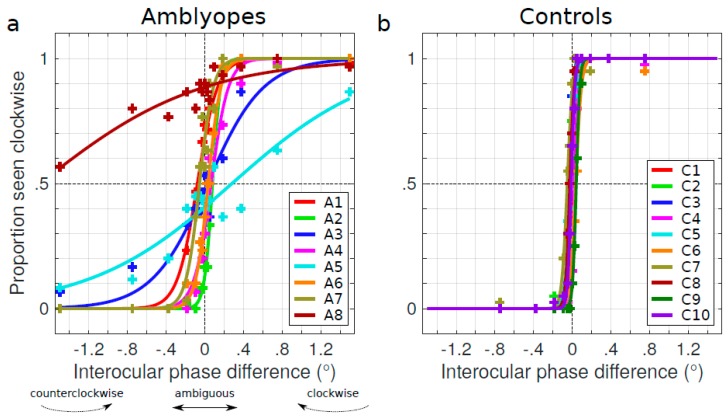
(**a**) Psychometric functions of the perceived direction as a function of the interocular phase difference for all eight amblyopic subjects. The midpoint of the logistic function at 0.5 performance defines the point of subjective equality (PSE). (**b**) Psychometric functions of the perceived direction as a function of the interocular phase difference for the control subjects from our previous article [24].

The data were analyzed with Matlab R2017b (© the MathWorks). Figure 2 depicts the psychometric functions of the subjects reporting a clockwise perception as a function of the interocular phase difference. The psychometric functions were fitted with a logistic function forced between 0 and 1 (nonlinear least-squares regression, Matlab’s nlinfit function). The estimated midpoint of the logistic function defines the point of subjective equality (PSE), the point at which ambiguous motion in plane is perceived with a report proportion of 0.5 clockwise and 0.5 anticlockwise. This PSE was then rectified to allow comparisons of the effects regardless of which eye was amblyopic of each subject. The rectified point of subjective equality (rPSE) was computed as the actual PSE for subjects whose amblyopic eye was their left eye and its opposite for subjects whose amblyopic eye was their right eye (A1 and A2, see Table 1). Hence, a negative rPSE would mean that the non-amblyopic eye (NAE) was advanced and a positive one would mean that the NAE was delayed. Given the known rotation speed of the cylinder, the rPSE could be converted into interocular delay (IOD) between the amblyopic eye and the non-amblyopic eye (Equation (1)). The interocular delays for the base condition are reported for all amblyopic subjects in Table 1.

IOD = rPSE/ω,(1)

where ω is the angular speed of the stimulus.

## 3. Results

### 3.1. Characterizing the Spontaneous Pulfrich Phenomenon

We firstly characterized the spontaneous Pulfrich phenomenon that mild amblyopes experience. We analyzed the psychometric functions of amblyopic subjects reporting a clockwise perception of the direction of motion of the cylinder as a function of the interocular phase difference (Figure 2a). We can see here that, for some amblyopic subjects, this psychometric function is offset from 0°. This offset corresponds to the interocular delay (IOD) for each participant reported in Table 1 (see Section 2). All of these delays were significant (for each participant, the PSE was different from 0; *t*-test on the bootstrapped estimates of the PSE, α = 0.05). Surprisingly, these delays did not always affect the non-amblyopic eye (negative values). The amblyopic eye exhibited a negative delay in only three out of our eight participants (Table 1). Because the delays could affect either the non-amblyopic eye or the amblyopic eye of amblyopes, the average delay for the whole amblyopic group between the amblyopic and non-amblyopic eye was not significantly different from zero (two-sided Wilcoxon signed rank test, *W* = 19, *p* = 0.95). However, when comparing the psychometric functions of the amblyopes to the control group from our previous study (Figure 2b [24]), the PSE of the amblyopic group was much more variable compared to that of the controls. Indeed, the absolute value of the interocular delay |IOD| in the amblyopic group, 16.17 ± 32.13 ms (|PSE| = 0.29 ± 0.58), was significantly longer than in the control group, 1.54 ± 0.68 ms (|PSE| = 0.03 ± 0.01) (one-sided Wilcoxon rank sum test, U = 109, *p* = 0.001), even excluding the outlier amblyopic subject A8.

**Figure 3:**
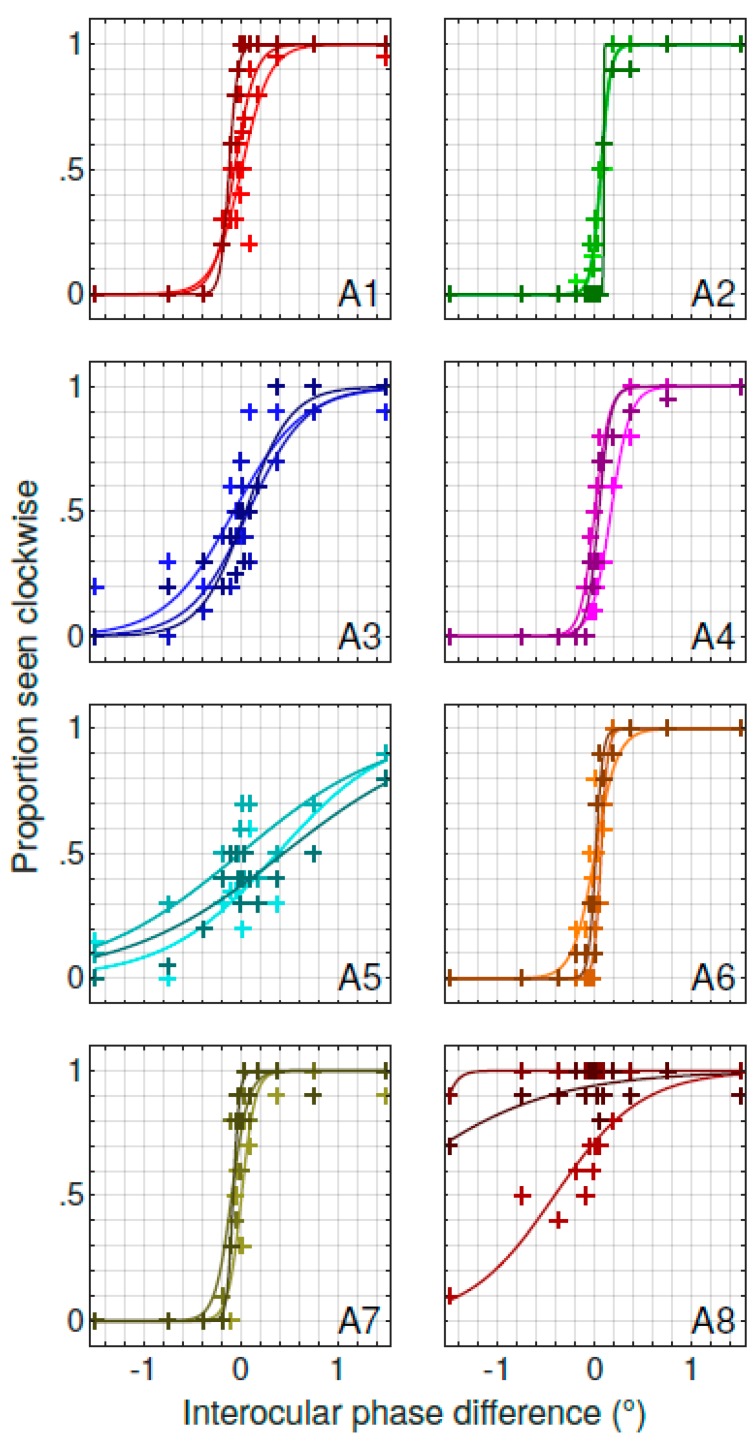
Variability of the PSE. The three repetitions (10 blocks each, light to dark shades) of the psychometric functions of the perceived direction of rotation as a function of the interocular phase difference for all amblyopic subjects (**A1**–**A8** in each panel). Data points are fitted by a logistic function.

In order to assess if the variability observed in the amblyopic group may be explained by an internal variability within each subject or if it reveals a consistent delay for each amblyopic subject that varies across the group of subjects, we compared three repeated measurements for each subject on different days in the base condition. The three measured psychometric functions, representing the perceived direction of rotation as a function of the interocular phase difference, are reported in Figure 3. It appears that the psychometric functions are quite similar from day to day, except for subject A8. To quantify the repeatability of this measure, we computed the intraclass correlation coefficient of the estimated PSE for the three repetitions for all subjects. It revealed a good correlation (*r* = 0.68) between the three repetitions, indicating that the IOD was consistent across sessions and, therefore, the variation that we observed previously was between, not within, amblyopes.

**Figure 4:**
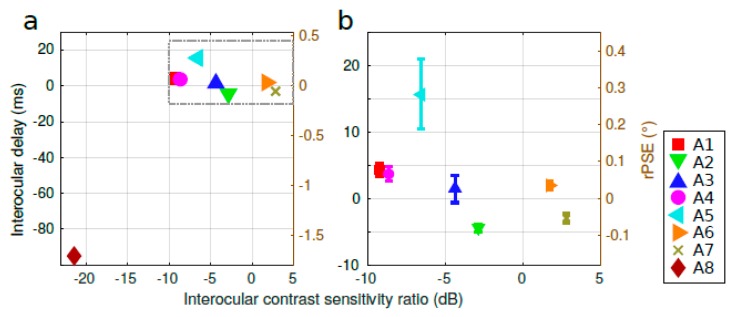
Correlation between the observed interocular delay and the interocular contrast sensitivity ratio. (**a**) Interocular delay as a function of the interocular contrast sensitivity ratio at the same spatial frequency of the Gabor patches used in the stimulus for all subjects. Coefficient of determination *R*^2^ = 0.5641, *p* = 0.0317. (**b**) Inset of the dotted frame area of (**a**) excluding subject A8. Coefficient of determination *R*^2^ = 0.2677, *p* = 0.2343.

Initially, we wondered if this delay could be the consequence of the loss of sensitivity in the amblyopic eye. Indeed, a contrast reduction in one eye can retard the processing of the information coming from that eye at the neurophysiological [32,33,34] and behavioral levels [35,36]. In particular, we previously showed in observers with typical vision that such a contrast difference could induce an illusion of depth (i.e., the Pulfrich phenomenon) and shift the psychometric function measured with an identical stimulus [24]. Therefore, in order to test if the delay observed with the amblyopic subjects was associated with the lower contrast gain in their amblyopic eye, we report in Figure 4a the correlation between the observed interocular delay and their interocular contrast sensitivity ratio at the same Gabor spatial frequency used in the stimulus (see Appendix A). This correlation was significant (coefficient of determination *R*^2^ = 0.56, *p* = 0.03); however, it was mainly driven by the data of subject A8. Excluding him resulted in a coefficient of determination *R*^2^ = 0.27, *p* = 0.23 (Figure 4b). Given that, in this regression, most subjects (five out of eight) presented a positive value of the interocular delay (i.e., a delay in the non-amblyopic eye), we conclude that this correlation, while significant, may not be meaningful.

### 3.2. Influence of the Stimulus Parameters

Any measured delay could be fixed, for example, due to slower optic nerve transmission; thus, it would not depend on stimulus parameters. Alternatively, the delay could be the result of a longer neural integration in the processing of visual information, in which case it would likely show a stimulus dependence [23]. To determine the type of delay, we manipulated the density, the spatial frequency, and the speed of the Gabor patches seen by the two eyes.

**Figure 5:**
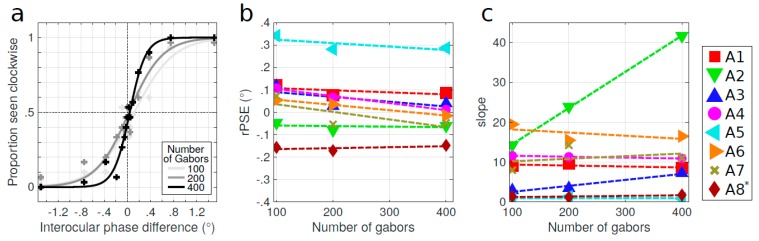
Effect of density. (**a**) Psychometric functions of the perceived direction of rotation for one representative amblyopic subject (A3) as a function of the interocular phase difference for three different numbers of Gabor patches composing the stimulus: 100 (light-gray symbols), 200 (mid-gray symbols), and 400 (dark-gray symbols). Data points are fitted by a logistic function. (**b**) Rectified PSE (rPSE) as a function of the number of Gabor patches for the eight amblyopic subjects. * rPSE values for subject A8 were divided by 10. (**c**) Slopes of the psychometric functions. Dashed lines represent linear regression. Quality of fit and regression parameters are reported in Table S1 (Supplementary Materials, Sheet 1).

An example is given in Figure 5a, where the psychometric functions of the perceived direction of rotation as a function of the interocular phase difference are displayed for one amblyopic subject. Results are shown for different numbers of Gabor patches defining the stimulus (100, 200, or 400). In this figure, it seems that the psychometric function shifted to the left and the PSE approached 0° when the number of Gabor patches was increased. The rPSE as a function of the number of Gabor patches defining the cylinder stimulus is then presented in Figure 5b for the eight amblyopic subjects. For most subjects, the rPSE approached 0° when more Gabor patches were presented. The data were fitted by linear regressions with slopes significantly converging, i.e., slopes were positive when the initial rPSE for 100 Gabor patches was negative, and they were negative when the initial rPSE was positive (one-sided Wilcoxon signed rank test, *W* = 1, *p* = 0.008). However, only two of these slopes were significant (see Table S1, Supplementary Materials). It should be noted that the rPSE values of subject A8 were scaled (divided) by a factor of 10 to be represented with other data. The slopes of the psychometric functions as a function of the number of Gabor patches are reported in Figure 5c. They did not show any consistent pattern, indicating that the density of the stimulus had no consistent effect on the quality of the performance in the task.

**Figure 6:**
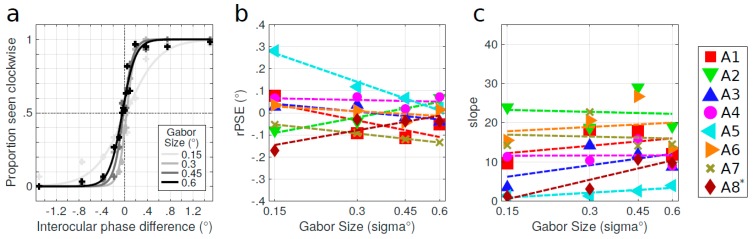
Effect of spatial frequency. (**a**) Psychometric functions of the perceived direction of rotation for one amblyopic subject (A3) as a function of the interocular phase difference for different sizes of the Gabor patches composing the stimulus: 0.15°, 0.3°, 0.45°, and 0.6° (from light gray to dark gray). Data points are fitted by a logistic function. (**b**) rPSE as a function of the size of the Gabor patches for the eight amblyopic subjects. * rPSE values for subject A8 were divided by 10. (**c**) Slopes of the psychometric functions. Dashed lines represent linear regression. Quality of fit and regression parameters are reported in Table S1 (Supplementary Materials, Sheet 2).

Secondly, in order to test the effect of the spatial frequency on the IOD, we manipulated the size of the Gabor patches which defined the stimulus. The psychometric functions of the perceived direction of rotation as a function of the interocular phase difference for one amblyopic subject are presented in Figure 6a. Results are shown for four different sizes of Gabor patches: 0.15, 0.3, 0.45, or 0.6°, which had spatial frequencies of 2.85, 1.43, 0.95, and 0.71 c/d, respectively. For these four sizes, the numbers of Gabor patches were respectively set to 200, 100, 66, and 50 in order to minimize overlap. The psychometric function shifted to the left and the PSE approached 0° when the size of the Gabor patches was enlarged (scaled). The rPSE as a function of the size of the Gabor patches is reported in Figure 6b for all amblyopic subjects. For most of them, the rPSE approached 0° when the size of the Gabor patches was increased, i.e., when the spatial frequency was decreased. This was indicated by the significantly converging slopes of the linear regressions (one-sided Wilcoxon signed rank test, *W* = 4, *p* = 0.03) although only three of these regression slopes were significant (see Table S1, Supplementary Materials). The slopes of the psychometric functions are reported in Figure 6c. The slopes significantly increased as a function of the size of the Gabor patches, which indicated that the psychometric functions were getting steeper (one-sided Wilcoxon signed rank test on the slopes of the linear regressions, *W* = 31, *p* = 0.04). This would indicate that participants’ performance got more reliable at low spatial frequency.

**Figure 7:**
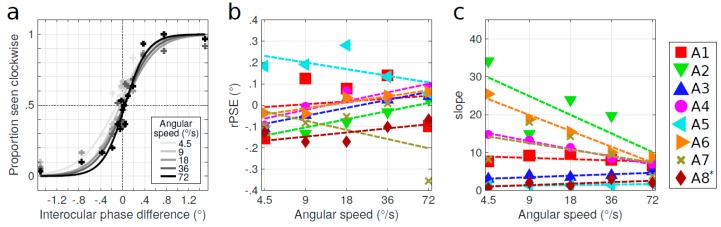
Effect of speed. (**a**) Psychometric functions of the perceived direction of rotation for one amblyopic subject (A3) as a function of the interocular phase difference for different rotation angular speeds of the stimulus: 4.5, 9, 18, 36, and 72 °/s (from light gray to dark gray). Data points are fitted by a logistic function. (**b**) rPSE as a function of the angular speed for the eight amblyopic subjects. * rPSE values for subject A8 were divided by 10. (**c**) Slopes of the psychometric functions. Dashed lines represent linear regression. Quality of fit and regression parameters are reported in Table S1 (Supplementary Materials, Sheet 3).

Finally, we manipulated the rotation speed of the cylinder stimulus. In Figure 7a, the psychometric functions for one amblyopic subject are displayed for different angular speeds: 4.5, 9, 18, 36, and 72°/s. The psychometric function shifted to the right and the PSE approached 0° when the rotation speed of the Gabor patches was increased. The rPSE of all subjects as a function of the angular rotation speed of the cylinder stimulus is presented in Figure 7c. For most subjects, the rPSE approached 0° as the rotation speed increased. However, the convergence of the slopes was not significant in this case because of the outlier data of subject A7, which was probably due to fusion issues at very high speed (all of her other data were aligned, describing a positive slope). This result was diagnostic because, at high speed, if there was a fixed transmission delay, one would expect that the resultant displacement and, hence, the equivalent phase would increase, not decrease as the data suggest. This observation suggests that it might be more reasonable to interpret the change in rPSE in terms of a longer neural integration time than delayed transmission per se. The slopes of the psychometric functions as a function of angular speed of the stimulus are reported in Figure 7c. The slopes which were initially shallow did not change much. However, the slopes which were initially steep decreased when the speed of the stimulus was increased. This indicates that the task was getting harder and less reliable at high speeds, which confirmed the irrelevance of the rPSE value of subject A7 at 72°/s.

## 4. Discussion

We confirm the observation made by Tredici and von Noorden [15] on three of their 70 patients that a spontaneous Pulfrich phenomenon exists in amblyopia. We developed a more sensitive method where we accurately measured the point of subjective equality as a function of stimulus disparity. Using this approach, we showed that the spontaneous Pulfrich phenomenon in amblyopia occurs in a significant percentage of patients and it depends on various stimulus parameters. Most notably, the observed dependence of the IOD on rotation velocity makes it more likely that the delay reflects changes in temporal integration within neurons rather than changes in transmission time between neurons. Also, the integration time within the amblyopic network was not always longer; sometimes, it was shorter, compared with that of the non-amblyopic eye stimulation. This resulted in a large variability in the basis for the spontaneous Pulfrich phenomenon between different amblyopic subjects.

Barbur et al. [21] also observed that, at low spatial frequency, the pupillary response to contrast gratings could be slightly faster in the amblyopic eye of anisometropes compared to their non-amblyopic eye. Nevertheless, our observation that in some cases the processing associated with the amblyopic eye could be faster than that associated with the non-amblyopic eye seems inconsistent with previous psychophysical studies on reaction times [16,23,37] and magnetoencephalography [38]. We speculate that this difference comes from the fact that these studies tested strong amblyopes, whereas we tested only mild amblyopes who exhibited a degree of stereopsis (mean acuity differences between the two eyes were respectively 0.8, 1.0, and 0.7 logMAR for Levi et al. (1979) [23], Loshin and Levi (1983) [37], and Chadnova et al. (2017) [38], compared to 0.38 logMAR in our cohort; data were unavailable in Hamasaki and Flynn (1981) [16]).

**Figure 8:**
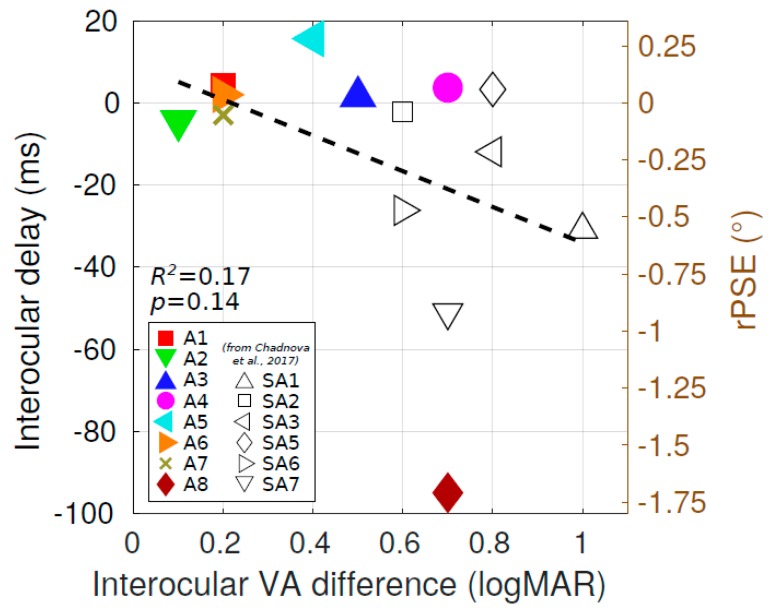
Correlation between interocular delay and interocular visual acuity (VA) difference in the amblyopic population. The delay observed in the amblyopic eye is plotted as a function of the difference in VA between the non-amblyopic eye (NAE) and the amblyopic eye (AE) for mild (filled color symbols) and strong (open black symbols) amblyopes. Data for strong amblyopes were taken from Reference [38]. The dashed line represents linear regression across the full population (coefficient of determination *R*^2^ = 0.17, *p* = 0.14; excluding subject A8: *R*^2^ = 0.22, *p* = 0.11).

Indeed, in a previous study using more severe amblyopes, we measured neural delays using frequency-tagged magnetoencephalography (MEG) [38]. In these more severe amblyopes, all the delays we measured involved the amblyopic eye function. For comparison, we combined the data from these two studies (the present one using psychophysics with mild amblyopes and the previous one using MEG with more severe amblyopes) to see if there was a correlation between the interocular delay (IOD) and the interocular visual acuity (VA) difference in the amblyopic population. The delay observed in the amblyopic eye is reported as a function of the difference in VA between the NAE and the AE for mild (this study) and strong (data from Reference [38]) amblyopes in Figure 8. It is, however, important to note that the IOD was assessed with two very different methodologies in the two studies. There seemed to be a trend of longer delays associated with larger interocular VA differences, although, for this sample size, this correlation was not significant (coefficient of determination *R*^2^ = 0.17, *p* = 0.14). This correlation slightly improved excluding subject A8 (*R*^2^ = 0.22, *p* = 0.11). A conclusion that there is a processing delay correlated with the degree of amblyopic deficit would receive some support from studies of reaction time differences to visual stimuli for amblyopic versus non-amblyopic eyes [16,23].

Our starting hypothesis was that, since we already demonstrated a contrast-dependent Pulfrich phenomenon in normals [24] (we replicated these results in amblyopes in Appendix B), any spontaneous Pulfrich phenomenon in amblyopes could be due to the known interocular difference in contrast sensitivity in amblyopia [39,40]. The finding that the polarity of the rPSE can differ between amblyopes rules this out as a general explanation. The same is true for any explanation that involves interocular suppression. The fact that there is only small intra-subject variability (i.e., the variability is between subjects not within subjects) rules out any explanation based on an instability in interocular timing. An alternative hypothesis could come from recent finding that blur induces a “reverse” Pulfrich phenomenon [41]. Indeed, in their paper, Burge et al. showed that removing the high spatial frequencies in one eye by optically blurring the image sped up the processing of that eye, resulting in the perception of a Pulfrich phenomenon in the direction of the blurred eye. In that case, the blur experienced by amblyopes in their amblyopic eye [42,43] could speed up the processing of the amblyopic eye and, thus, induce such reverse Pulfrich phenomena.

Hence, one possible explanation could be that the interocular timing difference could be governed by two components for the processing associated with the amblyopic eye: (i) a delay correlated with the strength of amblyopia, up to 156 ms [16], supported by the mild correlation we observe (Figure 8), and (ii) a modest acceleration, in the range of 4ms, due to blur [41]. For mild amblyopes, the blur-based acceleration might dominate, whereas, for more severe amblyopes, the dominant factor might be a neural processing delay associated with the amblyopic eye.

The observation that all but one subject had a negative rPSE at low speed (4.5°/s), while some of them switched their polarity at high speeds (Figure 7), could also find an explanation with this blur-based acceleration suggestion. Indeed, an object moving quickly might appear more blurred than an object moving slowly, thus generating a faster acceleration. It could also explain the decrease in the slope of the psychometric function at high speeds, which in that case could indicate a sort of position uncertainty.

One could argue that the fact that we changed the number of elements as we changed their size between the different spatial frequency conditions could have influenced the effect we observed. We cannot completely rule out this hypothesis. However, the density of the elements had only a mild effect on the PSE (mean absolute difference 0.07° between the minimum (100) and maximum (400) number of elements), whereas the spatial frequency had a much larger effect of 0.25° between the most extreme conditions with a similar ratio of 4:1 elements. Furthermore, such an effect of spatial frequency was not observed in a control population (see Appendix C). Moreover, while an increase in the number of elements reduced the absolute |rPSE|, The increase in the size of the elements, which also produced a reduction of the |rPSE|, was accompanied by a decrease in the number of elements. Therefore, the putative influence of the number of elements would have opposed the |rPSE| decrease observed in this condition, suggesting that the effect of spatial frequency might indeed be even bigger. On the other hand, the area covered by the Gabor patches increased with their size despite the decreasing number of elements. Hence, the absolute reduction in rPSE observed could be driven by spatial summation mechanisms. This effect of spatial frequency could be supported by the fact that, in amblyopia, the two eyes are more balanced at low spatial frequency [44,45,46,47]. The absolute |rPSE| might be reduced regardless of its initial polarity because the amblyopic delay might be reduced due to the eyes being more balanced, and the blur effect [41] might also be decreased due to blurring having less of an effect on low-spatial-frequency images.

It seems to us, on the basis of the stimulus dependence of the effect, that the spontaneous Pulfrich phenomenon experienced by a subgroup of amblyopes (those with mild amblyopia and some residual stereopsis) is likely caused by an alteration in temporal summation associated with the processing of information by the amblyopic visual system rather than a simple transmission delay [17,18,19,20]. This processing could involve two conflicting components: an “amblyopic delay” [16,23,38] and a “blur-based acceleration” [41]. In the aim of developing treatment procedures, if we want conditions under which the amblyopic eye is not put at a competitive disadvantage to that of the non-amblyopic eye (i.e., conditions that optimally favor binocular vision), then dynamic visual stimuli should be chosen to minimize not only the interocular contrast sensitivity difference but also the interocular delay difference. Stimuli of low spatial and high temporal frequency achieve both objectives.

## Figures and Tables

**Table 1:** Clinical details of amblyopic subjects. VA: visual acuity, strab.: strabismus, aniso.: anisometropia, NAE: non-amblyopic eye, AE: amblyopic eye, exo: exotropia, eso: esotropia, IOD: interocular delay. Suppression was measured with the Bagolini striated glasses test. Estimated interocular delay IOD is reported in the last column.

Subject	Age/Sex		Refraction	VA	Squint	Suppression	Randot (Arcsec)	Patching	Surgery	Type	IOD (ms)
A1	40/F	NAE (OS)	plano	20/16	R eso 5°	weak	60	no	no	strab.	4.28
		AE (OD)	plano	20/25							
A2	33/F	NAE (OS)	plano	20/12.5		weak central	100	no	no	aniso.	−4.41
		AE (OD)	+2.50	20/16							
A3	21/M	NAE (OD)	−2.75	20/10	L exo 5°	no	400	For 5 years	no	mixed	1.53
		AE (OS)	+1.75/−1.00 × 30°	20/32				around 11yo			
A4	25/F	NAE (OD)	−1.50	20/12.5		no	100	no	no	aniso.	3.69
		AE (OS)	+1.50	20/63							
A5	53/M	NAE (OD)	−1.25/−0.50 × 30°	20/20	L exo 6°	no	NA	no	no	mixed	15.62
		AE (OS)	+2.50/−1.50 × 75°	20/50							
A6	27/M	NAE (OD)	+1.50/−0.75 × 163°	20/12.5	L eso 5°	flickering	60	1 year	no	mixed	1.97
		AE (OS)	+3.50/−0.75 × 65°	20/20				around 5yo			
A7	54/F	NAE (OD)	plano	20/20	L eso 9°	weak	100	no	strabism surgery	strab.	−2.97
		AE (OS)	plano	20/32					6 months ago		
A8	32/M	NAE (OD)	−1	20/10	L eso 10°	weak	400	no	no	mixed	−94.93
		AE (OS)	+1.5	20/50							

**Figure A1:**
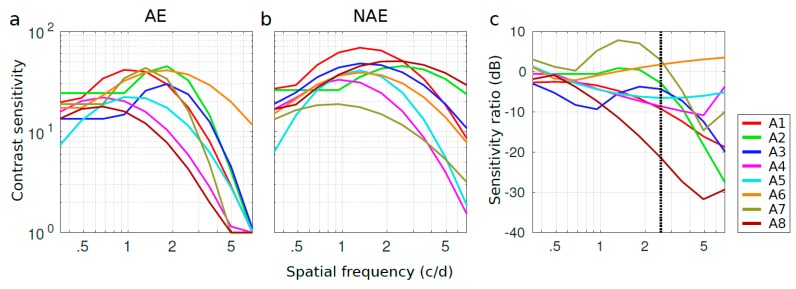
Contrast sensitivity of (**a**) the amblyopic eye (AE) and (**b**) the non-amblyopic eye (NAE) of all amblyopic subjects as a function of the spatial frequency. (**c**) Ratio between the monocular sensitivity AE/NAE as a function of the spatial frequency.

**Figure A2:**
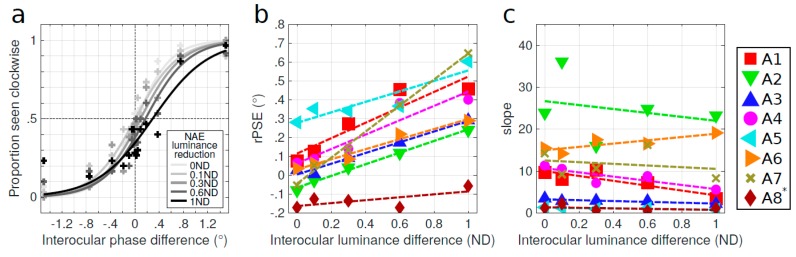
Interocular luminance difference. (**a**) Psychometric functions of the perceived direction of rotation for one representative amblyopic subject (A7) as a function of the interocular phase difference for five luminance conditions. Neutral density (ND) filters were placed in front of the non-amblyopic eye: no-filter, 0.1ND, 0.3ND, 0.6ND, and 1ND from light- to dark-gray symbols. Data points are fitted by a logistic function. (**b**) rPSE as a function of the interocular luminance difference for the individual eight amblyopic subjects. * rPSE values for subject A8 were divided by 10. (**c**) Slopes of the psychometric functions. Dashed lines represent linear regression. Quality of fit and regression parameters are reported in Table S1 (Supplementary Materials, Sheet 4).

**Figure A3:**
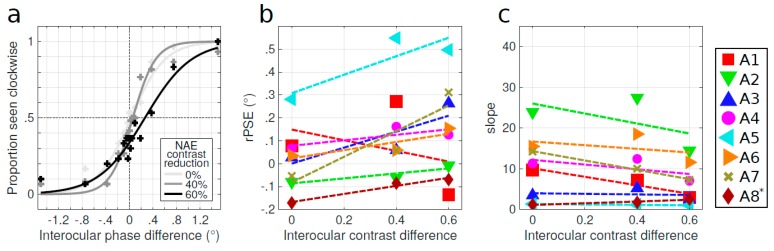
Interocular contrast difference. (**a**) Psychometric functions of the perceived direction of rotation for one amblyopic subject (A7) as a function of the interocular phase difference for three contrast conditions. Contrast was decreased in the non-amblyopic eye (NAE) image: 0% (light-gray symbols), 40% (mid-gray symbols), and 60% (dark-gray symbols) reduction. Data points are fitted by a logistic function. (**b**) rPSE as a function of the interocular contrast difference for the eight amblyopic subjects. *rPSE values for subject A8 were divided by 10. (**c**) Slopes of the psychometric functions. Dashed lines represent linear regression. Quality of fit and regression parameters are reported in Table S1 (Supplementary Materials, Sheet 5).

**Figure A4:**
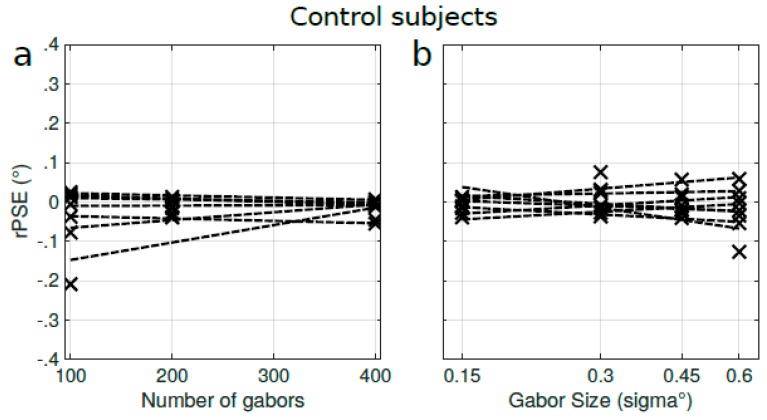
Effect of density and spatial frequency on control subjects. (**a**) rPSE as a function of the number of Gabor patches for eight control subjects. (**b**) rPSE as a function of the size of the Gabor patches for eight control subjects. Dashed lines represent linear regression.

## Supplementary Materials

The following are available online at https://www.mdpi.com/2411-5150/3/4/54/s1: Supplementary Videos 1, 2, and 3 show not-to-scale anaglyph examples of the stimulus presented with an interocular phase difference making the stimulus rotation ambiguous (Supplementary Video 1), clockwise (Supplementary Video 2), or counterclockwise (Supplementary Video 3). These videos can be viewed with standard red/green anaglyph glasses, with the red filter over the left eye. Supplementary Table S1 presents fit quality and regression parameters in all tested conditions. *R*^2^: coefficient of determination; MSE: mean squared error; a: regression slope; pf: *p*-value of the F-statistic; pt: *p*-value of the two-tailed *t*-statistic.

## Appendix A. Contrast Sensitivity of Amblyopic Subjects

The contrast sensitivities of the amblyopic eye (AE) and the non-amblyopic eye (NAE) of all amblyopic subjects as a function of the spatial frequency are presented in Figure A1a,b. They were measured with the quick contrast sensitivity function (qCSF) [48] with the same apparatus and procedure as used in Reference [44]. Then, the ratio between the monocular sensitivity AE/NAE was calculated; it is plotted as a function of the spatial frequency in Figure A1c.

## Appendix B. Contrast-Dependent Pulfrich Phenomenon in Amblyopia

The interocular delay can be manipulated by reducing the luminance or contrast seen by one eye in normal observers [24,49]. Thus, we then wanted to test if the delay observed in the amblyopic group could be compensated for by reducing the luminance or contrast in the non-amblyopic eye. An example is given in Figure A2a. The psychometric functions of one amblyopic subject who reported a clockwise perception as a function of the interocular delay for five luminance conditions are displayed, where an ND filter was placed in front of the non-amblyopic eye: no-filter, 0.1ND, 0.3ND, 0.6ND, and 1ND. One can see that the psychometric function shifted to the right when the luminance was decreased over the non-amblyopic eye (NAE), meaning that the rotation was seen more anticlockwise.

The estimated rPSE for changes in the interocular phase is then plotted as a function of the interocular luminance difference between the non-amblyopic and the amblyopic eyes in Figure A2b for all subjects. It should be noted that the rPSE values of subject A8 were scaled (divided) by a factor of 10 to be represented with other data. When an ND filter was placed in front of the NAE, the rPSE increased for all subjects, meaning that the processing of the information coming from the NAE was retarded by the luminance reduction. The data were fitted by linear regressions in a fashion similar to Carkeet et al. [49]. The estimated slopes were significantly positive for all subjects (mean slope 0.42 ± 0.20, one-sided Wilcoxon signed rank test, *W* = 36, *p* = 0.004). On average, the rPSE increased from −0.17° when there was no luminance difference to 0.30° at 1ND interocular luminance difference, which falls in the same range as we previously observed in the normal population [24].

Thus, for participants whose rPSE was initially positive, meaning that their NAE was actually delayed compared to their AE, applying an ND filter in front of the NAE drove the rPSE away from 0° and aggravated the interocular delay (IOD). For subjects A2 and A7 whose rPSE was initially negative, meaning that their AE was delayed compared to their NAE, applying an ND filter in front of the NAE brought the rPSE close to 0° for small ND values, but at some point continued to increase it and drove it beyond 0° for high luminance reductions, as can be observed by the continuous shift to the right of the psychometric function with increasing ND values in Figure A2a. Subject A8 presented an initial rPSE negative value so large that even the largest tested luminance reduction did not bring his rPSE to 0°. All of these results indicate that the reduction of luminance to one eye results in slowing down the processing of the information coming from that eye and is independent of the initial polarity of the delay.

The slopes of the psychometric functions as a function of the interocular luminance difference are presented in Figure A2c. Most of them decreased and this decrease was significant, which indicates that the task was getting harder and less reliable at high luminance differences (one-sided Wilcoxon signed rank test on the slopes of the linear regressions, *W* = 5, *p* = 0.04).

We showed previously on normals that a contrast reduction in one eye could induce a delay in the processing of the signal coming from this eye [24]. Hence, we wanted to assess if this effect was reproducible in the amblyopic population. Hence, we reduced the contrast of the Gabor patches seen by the non-amblyopic eye. This is actually a typical manipulation that reduces the interocular suppressive imbalance that amblyopes exhibit when viewing with both eyes open.

In Figure A3a, the psychometric functions are displayed for one amblyopic subject who reported a clockwise perception as a function of the interocular delay for three contrast conditions where the contrast of the Gabor patches was reduced in the image seen by the NAE: no reduction, 40% reduction, and 60% reduction. One can see that the psychometric function was shifted to the right when the contrast was decreased in the non-amblyopic eye image, meaning that the rotation was seen more anticlockwise. The estimated rPSE for changes in the interocular phase is then plotted as a function of the interocular contrast difference in Figure 4b for all subjects. Here again, the rPSE values of subject A8 were scaled by a factor of 10 to be represented with other data.

When the contrast of the Gabor patches seen by the NAE was decreased, the rPSE on average increased from −0.17° at 0% to 0.06° at 60% interocular contrast difference (Figure A3b). The data were fitted by linear regressions with significantly positive slopes (mean slope 0.41 ± 0.60, one-sided Wilcoxon signed rank test, *W* = 32, *p* = 0.03) despite one subject (subject A1) presenting a negative slope. This negative slope was due to his negative rPSE at 60% contrast difference, which we think is probably related to a visibility or fusion issue when the contrast is reduced too much, rather than an actual switch in the interocular delay. These estimated slopes of the rPSE as a function of the interocular contrast difference were significantly higher than in the normal population (one-sided Wilcoxon rank sum test, U = 73, *p* = 0.08; normal population data from Reference [24]). This comparison was still significant if the outlier amblyopic subjects A1 and A8 were excluded from the analysis and led to a slope approximately 2.5 times higher than for normals.

The slopes of the psychometric functions as a function of the interocular contrast difference are presented in Figure A3c. Most of them decreased and this decrease is significant, which indicates that the task was getting harder and less reliable when the contrast difference was severe (one-sided Wilcoxon signed rank test on the slopes of the linear regressions, *W* = 3, *p* = 0.02).

Similar to the results above for interocular changes in luminance, for participants whose rPSE was initially positive, reducing the contrast of the Gabor patches presented to the NAE drove the rPSE away from 0° and aggravated the IOD. For subjects whose rPSE was initially negative, meaning that their AE was delayed compared to their NAE, reducing the contrast of the image seen by the NAE brought the rPSE close to 0°. It even continued to increase it and drove it away from 0° for subject A7 for large contrast reductions, as can be observed by the continuous shift to the right of the psychometric function with decreasing contrast in Figure A3a. All of these results suggest that the contrast reduction results in a slowing down of the processing of the information coming from the eye independent of the initial polarity of the delay, which is comparable to what was previously described for changes in interocular luminance.

As we previously observed in controls, a monocular reduction of luminance or contrast in the input received by the non-amblyopic eye would slow down its processing, independently of the initial delay. Thus, it could compensate for or aggravate any spontaneous interocular delay observed in amblyopia. In controls, we previously observed a correlation between the regression slopes of the angular PSE as a function of the interocular contrast difference and the slopes of the angular PSE as a function of the interocular luminance difference [24]. However, for amblyopes, this same correlation using the rPSE was only significant because of the outlier subject A8 (coefficient of determination *R*^2^ = 0.5563, *p* = 0.0336); without his data, the coefficient of determination dropped to *R*^2^ = 0.1037 with *p* = 0.4812.

## Appendix C. Effect of Density and Spatial Frequency on Control Subjects

In Figure A4a are presented the rectified points of subjective equality (rPSE) of eight control subjects (including the two authors) as a function of the number of Gabor patches (with size 0.15° and spatial frequency 2.85 c/d). For control subjects, the rPSE was computed as the actual PSE for subjects whose dominant eye was their right eye and its opposite for subjects whose dominant eye was their left eye. The rPSE values were much less variable than in the amblyopic population and significantly converged when the number of Gabor patches was increased (one-sided Wilcoxon signed rank test, on the slopes of the linear regressions, *W* = 4, *p* = 0.03). In Figure A4b are presented the rPSE values of the control subjects as a function of the size of the Gabor patches: 0.15, 0.3, 0.45, or 0.6°, which had spatial frequencies of 2.85, 1.43, 0.95, and 0.71 c/d respectively. For these four sizes, the number of Gabor patches was respectively set to 200, 100, 66 and 50. Here again, the rPSE values were much less variable than in the amblyopic population, particularly at high spatial frequencies (small sizes). Conversely to what is observed with amblyopes, for control subjects, it seems that the rPSE became more variable when the spatial frequency of the Gabor patches was decreased, characterized by a divergence in the rPSE as a function of the size of the patches. However, this divergence was not significant (one-sided Wilcoxon signed rank test, on the slopes of the linear regressions, *W* = 19, *p* = 0.47).
